# Interleaved and simultaneous multi‐nuclear magnetic resonance in vivo. Review of principles, applications and potential

**DOI:** 10.1002/nbm.4735

**Published:** 2022-04-27

**Authors:** Alfredo L. Lopez Kolkovsky, Pierre G. Carlier, Benjamin Marty, Martin Meyerspeer

**Affiliations:** ^1^ NMR Laboratory, Neuromuscular Investigation Center Institute of Myology Paris France; ^2^ NMR laboratory CEA, DRF, IBFJ Paris France; ^3^ High‐Field MR Center, Center for Medical Physics and Biomedical Engineering Medical University of Vienna Vienna Austria

**Keywords:** interleaved, MRI, MRS, multi‐nuclear, simultaneous, X‐nucleus

## Abstract

Magnetic resonance signals from different nuclei can be excited or received at the same time,rendering simultaneous or rapidly interleaved multi‐nuclear acquisitions feasible. The advan‐tages are a reduction of total scan time compared to sequential multi‐nuclear acquisitions or that additional information from heteronuclear data is obtained at thesame time and anatomical position. Information content can be qualitatively increased by delivering a more comprehensive MR‐based picture of a transient state (such as an exercise bout). Also, combiningnon‐proton MR acquisitions with ^1^Hinformation (e.g., dynamic shim updates and motion correction) can be used to improve data quality during long scans and benefits image coregistration. This work reviews the literature on interleaved and simultaneous multi‐nuclear MRI and MRS in vivo. Prominent use cases for this methodology in clinical and research applications are brain and muscle, but studies have also been carried out in other targets, including the lung, knee, breast and heart. Simultaneous multi‐nuclear measurements in the liver and kidney have also been performed, but exclusively in rodents. In this review, a consistent nomenclature is proposed, to help clarify the terminology used for this principle throughout the literature on in‐vivo MR. An overview covers the basic principles, the technical requirements on the MR scanner and the implementations realised either by MR system vendors or research groups, from the early days until today. Considerations regarding the multi‐tuned RF coils required and heteronuclear polarisation interactions are briefly discussed, and fields for future in‐vivo applications for interleaved multi‐nuclear MR pulse sequences are identified.

## Graphical abstract

MR signals from different nuclei can be excited or received at the same time, rendering simultaneous or interleaved multi‐nuclear acquisitions feasible. This has been used in vivo to reduce the total scan time, to acquire multi‐parametric data during a transient physiological state or to improve non‐proton MR data quality with ^1^H navigators. The principles and the technical implementation of this technique, from the early days until today, are presented, and clinical and research applications are discussed.

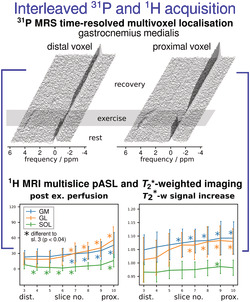

**Abbreviations**
ASL: arterial spin labelling
ATP: adenosine triphosphate
BOLD: blood oxygenation level dependent
CA: contrast agent
CBF: cerebral blood flow
CMRO_2_
: cerebral metabolic rate of oxygen consumption
dMb: deoxymyoglobin
DQF: double quantum filtered
G6P: glucose‐6‐phosphate
GRE: gradient echo
HP: hyperpolarized
Lac: lactate
Mb: myoglobin
MRSI: magnetic resonance spectroscopic imaging
nOe: nuclear Overhauser effect (or enhancement)
PCr: phosphocreatine
PD: proton density
P_i_
: inorganic phosphate
PO_2_
: oxygen partial pressure
SAR: specific absorption rate
SNR: signal‐to‐noise ratio
SVS: single‐voxel spectroscopy
T/R switch: transmit/receive switch
UTE: ultra‐short $T_{E}$
ZTE: zero echo time

## 1 INTRODUCTION

MRI has become a major diagnostic tool in medical routine, and the main application modality of magnetic resonance in vivo. Beyond this, MRS has been established as a clinical research tool for brain disorders.^1^ The vast majority of these NMR applications performed today are based on exciting the magnetic moment of hydrogen (^1^H) nuclei. However, the application of NMR is not limited to ^1^H, as other physiologically relevant nuclei (e.g., ^2^H, ^3^He, ^13^C, ^17^O, ^19^F, ^23^Na, ^31^P or ^129^Xe) give rise to an NMR signal, but are less abundant and intrinsically less sensitive than ^1^H.

Such non‐proton or ‘X‐nucleus’ studies can provide complementary information not available from ^1^H NMR. For example, phosphorus‐31 (^31^P) MRS has been employed as a tool to investigate intracellular pH and metabolism in vivo since its early days^2^, ^3^, ^4^ and continuously throughout, particularly in skeletal muscle.^5^, ^6^ It has proven useful to study metabolites in liver,^5^, ^7^ heart and brain^8^ as well as bone mineralization^9^, ^10^ and oncology^11^, ^12^; carbon‐13 (^13^C) can provide information about the metabolism of glucose and glycogen in vivo^4^, ^13^, ^14^; deuterium‐2 (^2^H) is also suited to evaluate glucose metabolism^15^, ^16^, ^17^ and as a tracer^18^, ^19^; fluorine‐19 (^19^F) for cell tracking, monitoring of fluorinated drugs and as an alternative to hyperpolarized (HP) helium‐3 (^3^He) and xenon‐129 (^129^Xe) gases in functional lung imaging and ventilation studies^20^, ^21^, ^22^ or oxygen‐17 (^17^O) to image and quantify the metabolic rate of oxygen consumption and as a tracer of cerebral blood flow (CBF).^23^, ^24^ The viability of healthy and tumorous tissue can be studied with sodium‐23 (^23^Na) imaging and spectroscopy,^25^ which is also a valuable tool for the diagnosis and research of kidney^25^ and cartilage defects.^26^

Often ^1^H and X‐nuclear MR data from the same subject are required, for instance to correlate high‐resolution anatomic ^1^H images with metabolic information from X‐nuclear MR or to confront different types of functional information based on different nuclei. Acquiring these datasets sequentially has several disadvantages. Most obviously, the acquisition time adds to the (costly) total scan time, with negative bearing on the subject's comfort and cooperation. However, also comparison of datasets acquired during transient stimuli is hampered with sequential acquisitions because the stimulation and response may not be strictly reproducible as such; additionally, repeated stimulation may have undesired effects (e.g., fatigue or habituation). Data requiring an identical anatomical position, such as ^1^H, ^3^He or ^129^Xe images during ventilation studies, may also be challenging to obtain over separate breath‐holds. Furthermore, with sequential acquisitions, X‐nucleus MR cannot benefit from real‐time adjustments derived from ^1^H MRI, such as navigators or dynamic shim updates.

Fortunately, the Larmor frequencies of the pertinent nuclei are at least several hundred kilohertz apart at clinically relevant field strengths and it is therefore possible to independently excite and receive signals from different nuclei at the same time. This allows for multi‐nuclear acquisitions in a single scan by collecting data of each nucleus either truly simultaneously or in rapidly interleaved acquisitions.

The feasibility of the approach, which can help overcome the disadvantages of sequential measurements, was demonstrated by Thulborn et al^27^ as early as 1981 and was then employed in several pioneering works in humans.^28^, ^29^ The potential of reducing total measurement time was demonstrated in various studies^30^, ^31^ and the possibility to obtain multiple datasets in a single measurement has been exploited by acquiring complementary data from transient states that are problematic to repeat precisely—for example, in exercising muscles,^32^, ^33^, ^34^ during hypocapnia^35^ or in ventilation studies.^36^, ^37^

NMR sensitivity increases with magnetic field strength,^38^, ^39^ motivating the trend towards higher $B_{0}$ fields. The field strength of 3 T is becoming the standard for clinical scanners, while 7 T and above are becoming more widespread for research systems.^40^, ^41^, ^42^ This development is particularly interesting for non‐proton MR, as X‐nuclear MR examinations are now feasible in clinically relevant scan times, providing more specific data at higher temporal or spatial resolution than at lower fields. Consequently, the increasing availability of high‐field MR scanners has renewed the interest in non‐proton MR in general,^6^, ^24^, ^26^, ^42^ a key prerequisite for simultaneous and interleaved multi‐nuclear MR. The off‐the‐shelf hardware support of interleaved multi‐nuclear measurements in modern clinical scanners has also contributed to the latest increase of interleaved applications.

In this review, a consistent terminology is proposed, in line with the literature on interleaved and simultaneous multi‐nuclear MR in vivo. Technical obstacles and solutions are discussed, as well as the main applications and their advantages over conventional, sequential acquisition. Dual‐tuned RF coils, necessary for multi‐nuclear measurements, and potential heteronuclear interactions, such as nuclear Overhauser enhancement (nOe), are briefly discussed. Some perspectives for clinical applications using interleaved measurements are indicated to conclude this review.

## 2 TERMINOLOGY

**FIGURE 1:**
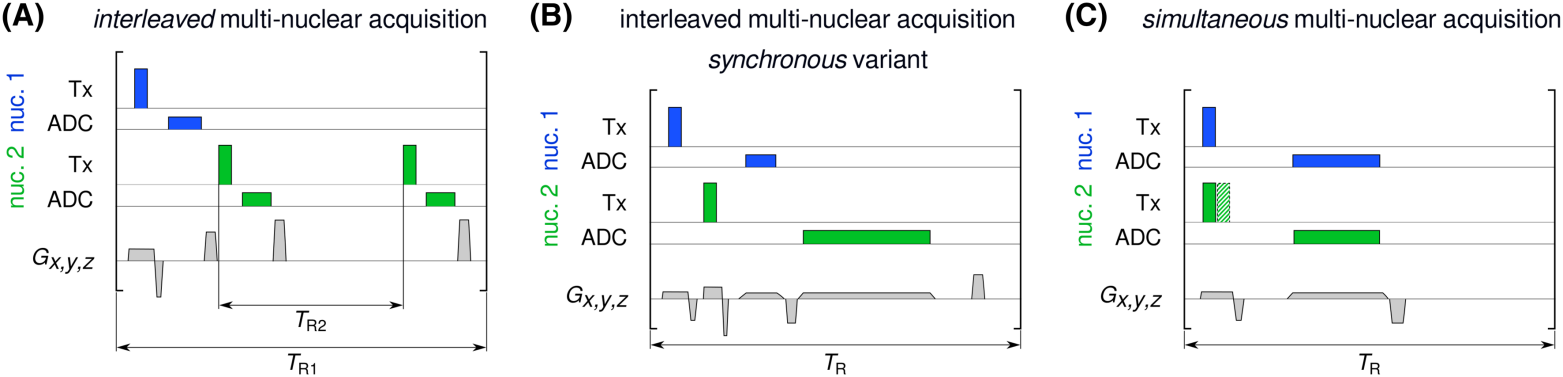
Illustrative schemes of RF transmission (Tx), MR signal recording (ADC) and magnetic field gradients ( $G_{x,y,z}$) during interleaved and simultaneous multi‐nuclear acquisitions of two nuclei (MRS or MRI). A, In interleaved sequences, data acquisition takes place sequentially for each nucleus, and different repetition times per nucleus ( $T_{R1}$ and $T_{R2}$, for Nuclei 1 and 2, respectively) are possible. For $T_{R}$ values to be constant throughout longer acquisitions, the ratio $T_{R1}$ : $T_{R2}$ must be integer. B, Alternatively, RF pulses and gradients can be interspersed (synchronous variant). C, For simultaneous acquisitions, excitation can be performed simultaneously or consecutively (hatched RF pulse of Nucleus 2) with a short delay required for switching. Note that in this example the slice‐selective gradient will simultaneously define the excitation slab thickness for both nuclei (together with the RF pulse profiles), while the frequency‐encoding gradient (together with readout bandwidths, set via dwell time) will set the respective fields of view in the read‐out direction

The topic of this review is the acquisition of datasets from different nuclei simultaneously or in close succession within a pulse sequence, in vivo. In agreement with the literature in this field, we suggest some consistent definitions:

- *Multi‐nuclear*: describes acquisitions with more than one type of NMR‐visible nucleus. This commonly refers to ^1^H and another nucleus, but combinations without ^1^H (References^43^, ^44^, ^45^) or with three to four different nuclei^37^, ^46^, ^47^ have been realized.
- *Non‐proton*or *X‐nucleus*: designates MR measurements with any nucleus other than ^1^H.
- *Interleaved*: a multi‐nuclear measurement is considered ‘interleaved’ when different datasets are acquired sequentially within a short time, typically within the repetition time $T_{R}$ of a pulse sequence. The criterion is that the signal of only one type of nucleus is received at a time (Figure 1). The sequence elements (i.e., RF and gradient pulses) for the different nuclei are played out either consecutively without mutual overlap (Figure 1A) or interspersed before data are sampled, still consecutively for each dataset (Figure 1B). This latter variant has been termed ‘synchronous’ acquisition.^48^
- *Simultaneous*: multi‐nuclear datasets can be acquired by receiving NMR signals of different nuclei truly simultaneously, that is, ADC sampling of signals with different resonance frequencies at the same time (Figure 1C).

It is worth stressing that the criterion for *multi‐nuclear interleaved* or *simultaneous* acquisition lies in the *reception* of the NMR signal and not in the RF transmission for different nuclei.

The term ‘interleaved’ is also used outside the context of multi‐nuclear MR: for example, for ^1^H imaging with different contrasts,^49^ parameters or slice positions^50^, ^51^; combining MRS sequences sensitive to different metabolites, voxel positions^52^, ^53^, ^54^ or with added editing pulses^32^, ^55^ or merging imaging and spectroscopy sequences into a single experiment.^56^, ^57^, ^58^ While such ‘interleaved’ techniques are not per se the topic of this review, they can be and have been combined with multi‐nuclear interleaved measurements.^32^, ^34^, ^55^, ^59^, ^60^

Finally, the terms ‘interleaved’ and ‘simultaneous’ have sometimes been used in the literature to describe measurements with different nuclei that were actually performed in consecutive scans^61^, ^62^ and not even necessarily in the same scan session.

Other terminology has been used, for example, occasionally ‘time shared’^63^ for ‘interleaving’, or ‘parallel’^64^ for ‘simultaneous’. The latter is common terminology for high‐resolution NMR in liquids and solids, but is uncommon with in vivo literature (where it would conflict with, e.g., ‘parallel imaging’). Interleaved sub‐variants have also been defined for diverse polarization transfer and indirect detection methods, but these have not been applied in vivo.^64^

## 3 BENEFITS OF INTERLEAVED AND SIMULTANEOUS MULTI‐NUCLEAR MR

### 3.1 Scan time reduction

The most obvious advantage of multi‐nuclear interleaving is a reduction of the total scan time. Interleaved and synchronous measurements reduce the total duration by using the idle period of the first dataset acquisition for a second dataset acquisition. The waiting times present in $T_{1}$‐, $T_{2}$‐ or diffusion‐weighted imaging, during the post‐labelling delay of arterial spin labelling (ASL) measurements or simply to allow for longitudinal magnetization recovery are typical examples of idle periods suitable for secondary nucleus acquisitions. Also, different rates (i.e., different $T_{R}$ values) or MR signal recording times (i.e., the ADC sampling durations) can be implemented, taking into account the different relaxation times of nuclei or echo train lengths to optimize signal‐to‐noise ratio (SNR), while still reducing the total acquisition time.^28^, ^31^, ^65^, ^66^, ^67^, ^68^ Simultaneous multi‐nuclear acquisitions can further reduce the total sequence duration by overlapping the ADC recordings for the two nuclei, and may be particularly useful in measurements where little to no delay time is used, such as ^1^H and ^23^Na gradient echo (GRE) and ultra‐short $T_{E}$(UTE) MRI.^69^, ^70^

**FIGURE 2:**
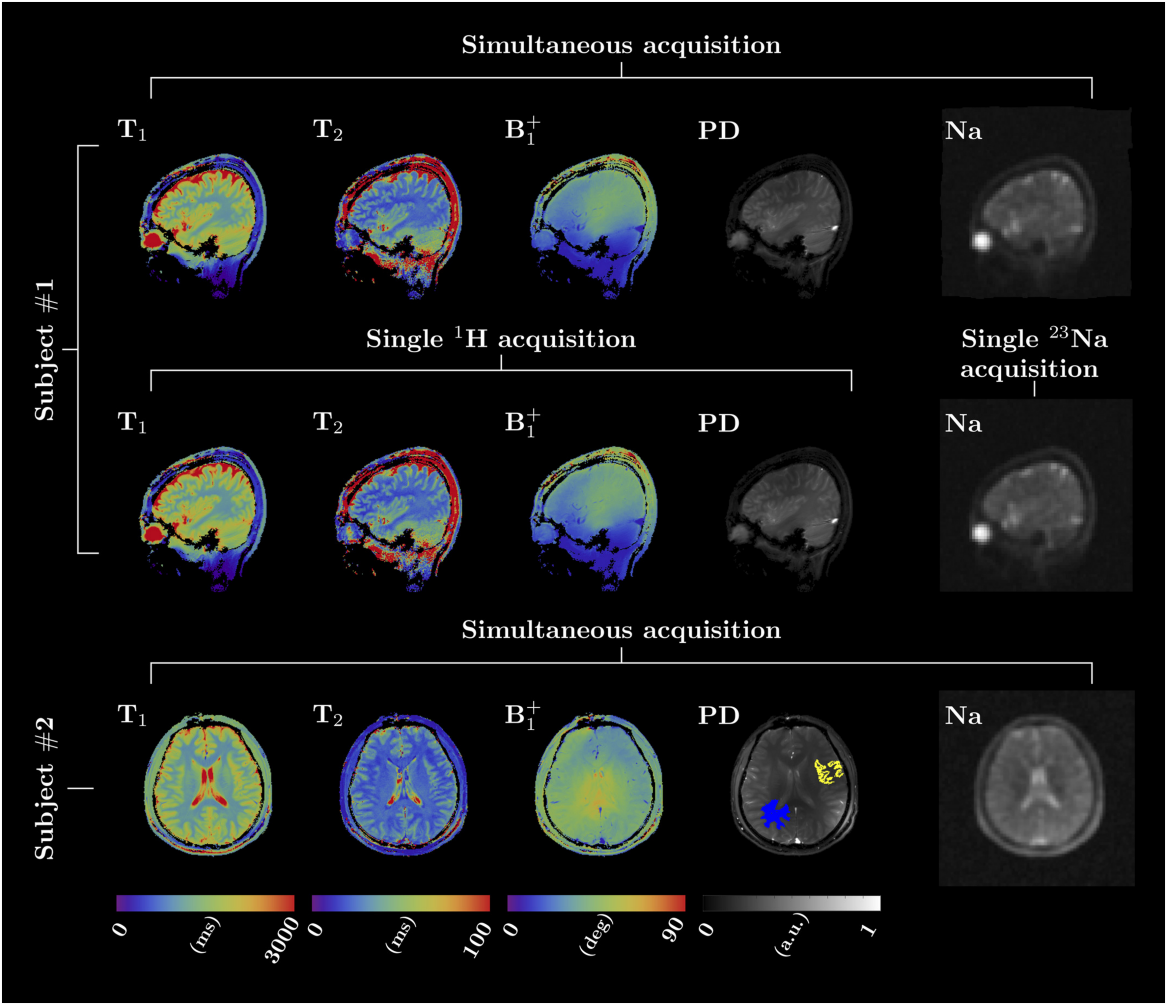
Proof of concept for simultaneously acquired ^23^Na MRI and ^1^H MR fingerprinting used to generate $T_{1}$, $T_{2}$, $B_{1}^{+}$ and PD maps of the human brain at 7 T on two healthy subjects. The sagittal images extracted from the simultaneous acquisition and the single‐nucleus scans are shown for Subject 1. Figure reproduced with permission from Reference^70^

Reducing the total acquisition time by interleaving has been achieved in the brain,^30^, ^65^, ^70^, ^71^ knee^31^, ^48^ and breast.^69^ In a recent work,^70^ simultaneous ^23^Na and ^1^H radial imaging was used to acquire ^1^H $T_{1}$, $T_{2}$, proton density (PD) and $B_{1}^{+}$ maps using MR fingerprinting and ^23^Na density images, at 7 T in the brain (Figure 2).

### 3.2 Multiparametric information

**FIGURE 3:**
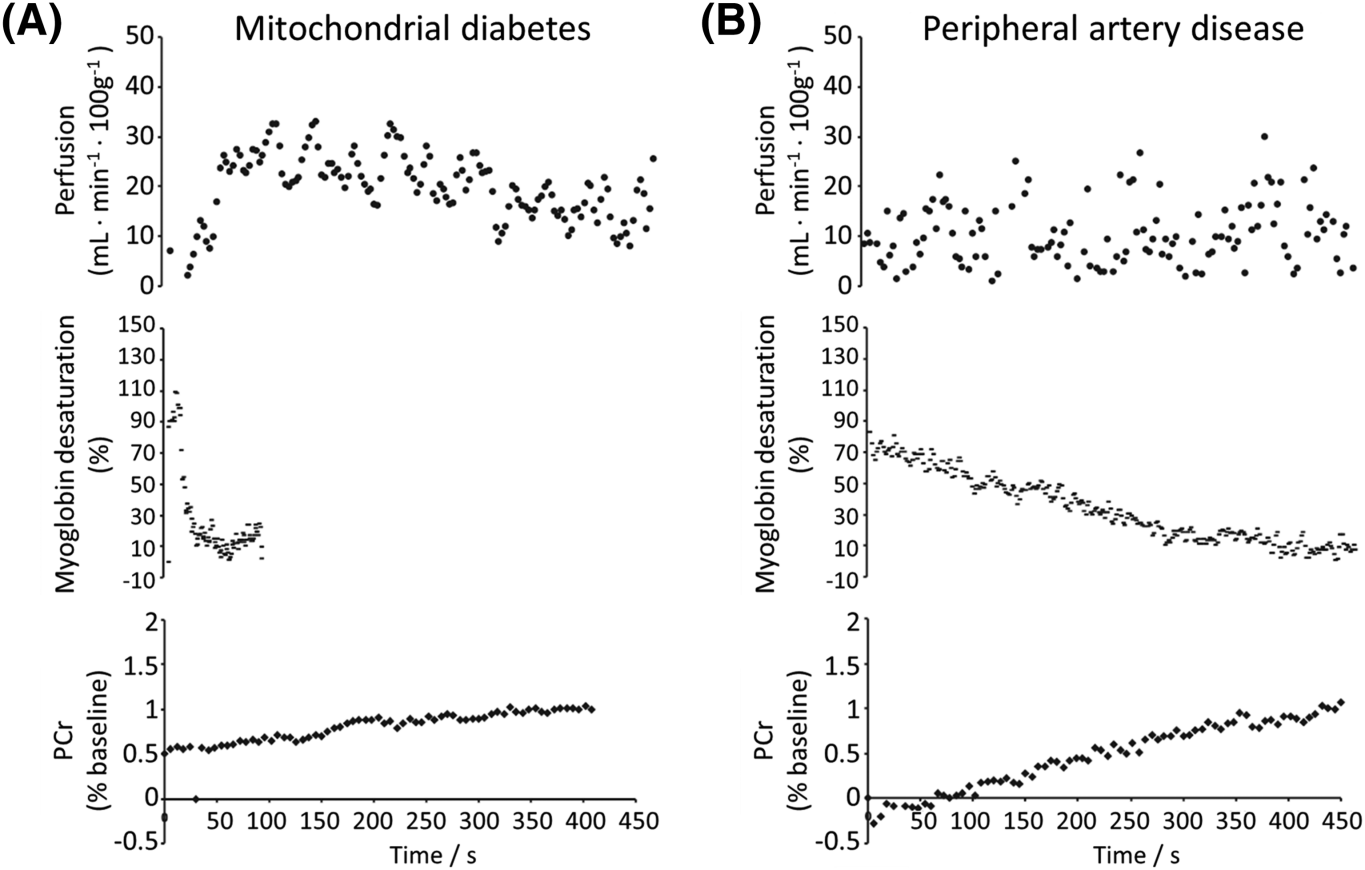
Examples of multiparametric functional NMR studies performed in a patient with mitochondrial diabetes (A) and another with peripheral artery disease (B). After a plantar flexion ischaemic bout, time curves of calf muscle perfusion (top), Mb resaturation (middle) and creatine rephosphorylation (bottom) were simultaneously monitored by interleaving ASL imaging and ^1^H and ^31^P NMR spectroscopy, respectively. In both conditions, the creatine rephosphorylation rate, an indicator of mitochondrial ATP resynthesis, was abnormally low. In B, mitochondrial dysfunction was clearly attributable to a blunted functional hyperaemia (top) and a dramatically slow muscle reoxygenation (middle). In A, post‐exercise reperfusion and Mb resaturation were within normal ranges, indicating an intrinsic defect of mitochondrial function^73^

Dynamic studies greatly benefit from acquiring multiple datasets simultaneously. Through interleaving, a single transient test can generate complementary information that can be readily combined to extract multiparametric biological variables that would be challenging to calculate otherwise.^37^, ^72^ During an MR examination, the physiological response to a dynamic stimulus or precise lung inflation state may be difficult to reproduce. In certain cases the test might even be impossible to repeat in the same examination, notably following the injection of contrast agent (CA) or with patients showing slow or compromised physiologic recovery. Other examples of functional paradigms outside the brain include exercise bouts, muscle ischaemia or the administration of tracers, drugs or enriched substrates to study their biodistribution, metabolism or pharmacokinetics. Furthermore, measuring multiple MR parameters during stimulation can reveal alterations within the probed concomitant biological processes that may otherwise not manifest in a basal state^73^, ^74^, ^75^ and show the temporal relationships between them.^32^, ^55^, ^76^ The technique is particularly interesting in pathologies where compensatory biological adaptations could be masking a failing physiological variable, misleading the clinical diagnosis. For instance, patient cases with abnormally low mitochondrial adenosine triphosphate (ATP) production have been characterized using a multiparametric sequence interleaving ^1^H imaging and spectroscopy with ^31^P MRS,^73^ with mitochondrial diabetes clearly distinguished from peripheral arterial disease by the normal perfusion and myoglobin (Mb) resaturation profiles (Figure 3). Other conditions can also be evaluated, such as the impact of ageing, physical training, nutritional supplementation,^77^ drugs and so forth.

### 3.3 Including dynamic ^1^H‐based adjustments and navigators

The quality of X‐nuclear MR data can potentially be improved by including dynamic adjustments derived from ^1^H MR. Examples are MR navigators,^78^ which can be used for prospective correction of respiratory and rigid bulk motion.^79^ Feedback‐based motion tracking and correction, $B_{0}$ shimming and frequency correction can increase the robustness of measurements^80^, ^81^, ^82^ and provide the means for real‐time quality control by rejecting or repeating data acquisitions compromised by motion.^83^, ^84^ This is particularly useful for pulse sequences where artefacts are difficult to detect or to correct (as in magnetic resonance spectroscopic imaging, MRSI), during exercise paradigms in the magnet where movement‐induced artefacts and $B_{0}$ variations are common or to alleviate examinations with patients experiencing difficulty in lying still. Fast ^1^H imaging can also be used for retrospective motion correction.^67^ Alternative non‐MR motion correction methods track rigid‐body movements only and require additional hardware.^79^, ^85^, ^86^, ^87^, ^88^, ^89^

An example is a cardiac MRS study in humans,^90^, ^91^ demonstrating ^1^H MR based volume tracking for compensation of respiratory motion to avoid contamination from chest wall and liver ^31^P MRS. Results from nine healthy volunteers measured at 1.5 T showed an average increase in fitting accuracy and signal amplitude with respect to the reference data.^90^ More recently, at 7 T, retrospective motion correction was applied to ^23^Na MRI of the human brain using interleaved ^1^H 3D navigator images,^92^ increasing the consistency between consecutive scans and improving the robustness of image quality against motion.

Motion correction has further been exploited in rodents, for X‐nucleus imaging of lung,^93^, ^94^, ^95^ heart^93^ and kidney.^67^

### 3.4 nOe, polarization transfer and ^1^H decoupling

Signal enhancement of low‐sensitivity nuclei, such as ^13^C, ^15^N, ^19^F or ^31^P, can be achieved by exploiting the heteronuclear spin–spin or dipolar coupling interactions with ^1^H nuclei by means of nOe,^96^, ^97^ polarization transfer^98^, ^99^, ^100^ or ^1^H decoupling.^97^ Although these methods can be applied without simultaneous or interleaved multi‐nuclear signal reception and are therefore not per se the core topic of this review, they are closely related and can be combined. NOe has been frequently observed with interleaved sequences, as it can be induced by the pulses for the ^1^H acquisition, even without addition of dedicated nOe pulses. Polarization transfer requires deliberate adjustment of flip angles and echo time, taking scalar coupling constants into account. Heteronuclear decoupling is achieved by transmitting on one Larmor frequency while receiving on the other, and hence still allows for interleaving but conflicts with simultaneous acquisition.

The nOe originates from the dipole interactions with the saturated ^1^H nuclei, with the effective enhancement value depending on numerous experimental aspects including magnetic field strength, ^1^H irradiation intensity, biological tissue type and physiological state.^96^, ^97^ The application of ^1^H decoupling, typically achieved using a WALTZ scheme^101^ during the X‐nuclei read‐out, collapses the split peaks of coupled resonances into singlets, greatly improving the sensitivity and simplifying spectral fitting. Decoupling pulses generate nOe by themselves but additional irradiation can be applied to achieve full nOe. During ^1^H polarization transfer, broadband RF pulses with appropriate phases and flip angles are played out simultaneously for both nuclei, enhancing the heteronuclear J‐coupled resonances while removing uncoupled ones. The X‐nuclei spectrum is thus simplified and the baseline is flattened. The sequence timings are chosen based on the J‐coupling constant of the resonance of interest.

Unfortunately, the ^1^H irradiation needed in these techniques will increase energy deposition and unavoidably impact the ^1^H equilibrium magnetization. Though this might not be a concern in interleaved sequences where the ^1^H signal is solely used for motion or frequency corrections, it could be a limiting factor when ^1^H SNR is critical (such as MRS) or lead to bias in acquisitions employing magnetization‐preparation modules (such as ASL or long‐ $T_{1}$‐mapping MRI). Conversely, unintended heteronuclear nOe can be generated by the application of on‐resonance RF‐intensive ^1^H pulses, which is typically the case of magnetization‐preparation modules or in sequences employing adiabatic pulses.^60^, ^71^, ^102^ Furthermore, a larger signal enhancement will result if the complete volume of interest of the X‐nucleus measurement is irradiated.^60^

While nOe can increase SNR and repeatability^103^ and provide biological information by itself,^104^ its magnitude will depend on the experimental setting but also on the tissue type and potentially on the pathological state.^97^ Therefore, similar nOe values between studies can be assumed only if the sequence parameters and experimental conditions are largely conserved. Acquiring reference data for nOe characterization will come at a cost in additional acquisition time, which will depend on the available X‐nucleus SNR and encoding scheme. Estimating nOe in preparatory measurements is recommended,^6^, ^96^ and will allow calculation of ‘nOe‐free’ metabolite concentrations and ratios.^60^

## 4 TECHNICAL REQUIREMENTS AND IMPLEMENTATIONS

### 4.1 Basic principles

Simultaneous multi‐nuclear acquisition offers true synchronicity of signal recording, at the cost of higher technical demands than interleaved acquisitions, and it causes dependences between acquisition parameters. While RF excitation and reception is independent between nuclei (setting aside heteronuclear polarization transfer, ^1^H decoupling and potentially nOe, as discussed above), magnetic field gradients always act on transverse magnetization and higher‐order spin coherences of all spin systems. The gradient trajectory being identical during simultaneous acquisitions leads to different fields of view for nuclei with different gyromagnetic ratios, which can be corrected for by k‐space regridding,^67^, ^70^ as a gradient‐linearity correction term to the MR system^94^ (when the gyromagnetic ratios are close, such as ^1^H and ^19^F) or, in principle, by setting the readout bandwidth per nucleus, via different ADC dwell times of separate receivers or by using sufficient oversampling. Similarly, simultaneous slice‐selective excitation or refocusing results in identical imaging slab orientations while the slice profiles and thicknesses are controllable via the RF pulse shape and bandwidth, which can be set individually for each nucleus. The alternative approach of interleaving multi‐nuclear acquisition relaxes the timing constraints to beyond the data acquisition duration (typically fractions of seconds) or repetition times. Interleaving can offer more flexibility with respect to field of view geometries, matrix sizes and repetition times, and even different types of acquisition scheme can be used: for example, combining ^1^H imaging and X‐nucleus spectroscopy.^32^, ^34^, ^72^

### 4.2 Requirements on the MR scanner

Several prerequisites on the MR scanner's hardware and software have to be met for multi‐nuclear interleaved or simultaneous acquisitions. The system must be able to transmit RF pulses at multiple resonance frequencies within one pulse sequence, either in rapid succession or simultaneously. This requires an RF transmit and receive system (including power amplifiers, multiple‐tuned RF coils, interfaces and the signal acquisition chain from preamplifiers to sampling hardware) that can operate at different Larmor frequencies and allows for rapid switching between nuclei within a pulse sequence. Finally, the pulse sequence and data processing pipeline (e.g., inline image reconstruction systems) have to be implemented so as to drive the RF pulses and to record and store the NMR signal at the required frequencies.

The challenge is that many systems, even when ready for measuring X‐nuclear data, are designed to acquire data of only a single nucleus within a pulse sequence. Most (clinical) MRI systems today are equipped with a power amplifier that can transmit RF within a narrow frequency band at the scanner's ^1^H frequency, sometimes wide enough for alternative ^19^F excitation. X‐nucleus excitation is usually achieved with an additional broadband amplifier, often with lower peak power and usually lower maximum output frequency than the ^1^H amplifier. The MR system's synthesizer frequency is mixed into the RF waveforms and then fed to the respective power amplifier. Transmission at two Larmor frequencies in one pulse sequence is fairly standard with X‐nucleus capable MR systems, for heteronuclear polarization transfer, nOe or indirect detection.^105^ Monitoring the specific absorption rate (SAR) is mandatory on human MRI systems and must therefore be readily implemented by the manufacturer, also for multi‐nuclear RF transmit. Therefore, no additional risk arises from using this capability for simultaneous or interleaved acquisitions. However, there is room for improvement in MR system and coil vendors' SAR management, which may often be too conservative because local SAR differs between ^1^H and X‐nuclei, and flip angle measurements are challenging with lower sensitivity. Unfortunately, systems capable of multi‐nuclear transmit cannot necessarily *receive* signals from different nuclei in one scan and may require hardware modifications in addition to the adaptations of pulse sequences and reconstruction pipelines. Handling the timing within the sequence, increased complexity (e.g., when parametrizing the protocol), additional data reconstruction steps and higher total SAR demand may also constitute additional challenges, depending on the application.

### 4.3 RF coils

RF coils are used to apply RF pulses and to receive the MR signals. On human systems with field strengths of up to 3 T, ^1^H transmit is commonly achieved with a body coil installed in the magnet bore, and X‐nucleus transmit is nearly always done with dedicated coils. Some systems disable the (^1^H transmit) body coil when a local transmit coil is plugged in, making dedicated dual‐frequency (X‐nucleus and ^1^H) local transmit coils obligatory for ^1^H and X‐nucleus RF measurements within the same examination. Body coils are not standard on ultra‐high‐field systems (although a ^31^P whole‐body coil has been presented at 7 T, Reference^106^, ^107^), and dedicated coils are generally used for all nuclei.

In principle, simultaneous and interleaved measurements are not limited by the RF coil itself, as long as it comprises channels for both Larmor frequencies. It may be necessary to adapt coil‐related software parameters, to allow the pulse sequence to activate the required transmit/receive (T/R) switches and preamplifiers at the necessary times. Further precautions should be taken, for example, to deal with transmission on one frequency while the preamplifier is active for the other, or to guarantee that this is avoided, to prevent hardware damage.

A practical difficulty of interleaved multi‐nuclear applications is the increased complexity (and cost) of dual‐tuned coils. Highly optimized ^1^H coils with a high channel count deliver maximum performance (high SNR, low mutual decoupling of elements and optimal placement for parallel imaging), but generally are proton‐only coils. Dual‐tuned coils are typically optimized for X‐nucleus sensitivity, with ^1^H elements designed for scout imaging and $B_{0}$ field mapping. They typically have a lower ^1^H channel count and inferior performance than coils optimized for ^1^H MR only,^108^, ^109^, ^110^, ^111^ which may limit the potential of interleaved and simultaneous multi‐nuclear applications. To improve the overall dual‐tuned coil performance and to allow for acquisition of high‐quality ^1^H data, innovative and organ‐specific coil designs have been developed.^37^, ^60^, ^112^, ^113^, ^114^, ^115^, ^116^, ^117^, ^118^, ^119^, ^120^, ^121^, ^122^

An in‐depth discussion on the trade‐offs for single‐ and multi‐structure dual‐tuned RF coils designs (focused on the brain but applicable to other anatomical targets) can be found elsewhere.^123^

### 4.4 Implemented MR system solutions for simultaneous and interleaved multi‐nuclear acquisitions

Simultaneous or interleaved signal reception has been realized in various ways by vendors and—in the early times of in vivo MR and later in cases where this was not possible on clinical MR scanners—by different research groups. The receiver of most MRI systems is based on the superheterodyne principle, that is, the signal is converted to an intermediate frequency^124^ of the order of a few megahertz, in one or several stages. The intermediate frequencies may or may not be different for different nuclei, according to the implementation by the manufacturer of the MR system.^125^ Hence, simultaneous or interleaved multi‐nuclear acquisitions may be possible straightforwardly (from the user perspective) or may necessitate hardware modifications.

An overview of the published implementation strategies for interleaved and simultaneous multi‐nuclear MR is given in Table 1, which is structured into three categories: (1) Early experimental MR systems built by commercial vendors or by the research groups, (2) MR imagers designed for clinical routine that require hardware modifications and (3) commercial MR scanners, on which this is possible without or with only minimal hardware modifications (e.g., rerouting cables) by the user.

**TABLE 1:** Published implementations of simultaneous and interleaved multi‐nuclear MR. The table is structured in three categories of hardware, representing early experimental systems, routine systems requiring hardware modifications and systems that support the techniques with only minimal or no hardware modifications

Category	Period	Manufacturer/model	Solution/challenges	References
Lab‐built or early experimental commercial systems	1981–1991	TMR, Oxford, Nicolet, Nalorac Cryogenics Corp, MIT/IBM	Additional spectrometer	^27^, ^63^, ^126^, ^127^
	1979, 1986, 1995	Custom built by lab	Switching receiver local oscillator frequency, separate transmitter and receiver	^28^, ^43^
	1983–1990, 1995	Bruker, Phospho‐energetics, Nicolet,GE	Frequency switching as implemented by constructor	^44^, ^45^, ^47^, ^128^
Scanner hardware modification by research group (with or without vendor support)	1994, 1996	Siemens SP63/GBS‐1	Additional spectrometer	^29^, ^30^, ^65^, ^71^
	1994–2000, 2021	Bruker	Modified RF switch (including transmit path), new electronic interface	^32^, ^66^, ^129^, ^130^, ^131^
	2006–2011	Philips Achieva	Modified spectrometer and software	^93^, ^94^, ^132^, ^133^, ^134^
	2011, 2013	Philips Achieva	Separate synthesizer and transmitter	^36^, ^37^
	2013–2020	Siemens Trio/Magnetom 7 T	Mix received signal or modify local oscillatorfrequency	^34^, ^48^, ^69^, ^70^, ^125^, ^135^
Hardware implementation by vendor	1999–2007	Bruker	MultiScan Control Tool	^59^, ^76^, ^136^, ^137^, ^138^, ^139^
				^55^, ^74^, ^77^, ^140^, ^141^
	2007–2015	Varian/Agilent	Rewiring, software modifications	^35^, ^67^, ^142^, ^143^
	2014–2020	Philips Gyroscan/Achieva/Ingenia	Software modifications	^31^, ^90^, ^95^, ^144^
	2016–2022	Siemens Prisma/Terra	Software modifications	^60^, ^92^, ^145^, ^146^, ^147^

The early works on multi‐nuclear interleaved and simultaneous measurements, particularly during the 1980s, profited from the research systems' relative openness of the hardware and software, that is, those systems (described in terms of ‘spectrometer and data processing system’ rather than ‘MR scanner’) required—and allowed—low‐level access to the hardware for operation. Solutions were to add spectrometers,^27^, ^126^ switches to alternate transmitters and receivers^63^ or, e.g., ‘simply changing the synthesizer frequency under computer control’ (see Schnall et al.,^128^). Several groups had designed custom‐built MR systems, foreseeing such capabilities.^28^, ^43^

Starting in the mid‐1990s, multi‐nuclear interleaved and simultaneous measurements were performed on large‐bore human MRI scanners, but because the capability was not implemented by the manufacturers this required custom hardware adaptations. Solutions involved auxiliary spectrometers^29^, ^71^, ^133^ or even an additional full RF transmit chain.^32^, ^36^, ^129^ An alternative approach is to shift either the local oscillator frequency of the superheterodyne receiver^125^ or the frequency of the received NMR signal itself.^135^ That is, to receive a second NMR signal, either the frequency of the local oscillator signal provided to the mixing stage in the receiver cassette is appropriately set, or the NMR signal's frequency is shifted using a mixer before being routed to the receiver. In both cases the resulting frequency at the digitization stage is what the system expects for acquisition of the default nucleus. This period saw declining publication activity in this field, which may well be a consequence of the technical and administrative difficulties arising from modifying the hardware of systems designed and certified for clinical applications.

Since 1999 and until today, vendors of pre‐clinical and human research systems have been offering hardware solutions allowing for interleaved or simultaneous multi‐nuclear MR. On clinical systems this became again possible without modifying the hardware about 10 years later, followed by a resurgence in publication activity involving human subjects after 2010. The vendor‐specific solutions (e.g., on the Bruker Avance, Siemens VD and upwards, and Philips Achieva platforms) generally involve one or several constant (i.e., independent of the nucleus) intermediate frequencies during signal reception in a superheterodyne receiver. Today, direct digitization of the NMR signal is implemented in the most recent hardware generations (e.g., Philips dStream technology), which in principle allows for acquisition of the signals of multiple nuclei at a time. Throughout all periods, simultaneous and interleaved techniques were used, though the majority of publications (about three in four) report on the latter approach.

## 5 CLINICAL AND RESEARCH APPLICATIONS

Interleaved and simultaneous acquisitions of multi‐nuclear MRI and MRS have been applied in clinical studies and research applications in human and animal studies. Table 2 gives an overview of these applications.

**TABLE 2:** Applications of interleaved (int), synchronous (syn) or simultaneous (sim) applications, sorted by studied organ, species and type of acquired data

Organ	Species	Sequences	Type	References
Muscle	Human	^1^H MRI + ^31^P MRS	int	^34^, ^77^, ^141^, ^144^
		^1^H MRI + ^1^H MRS + ^31^P MRS	int	^32^, ^60^, ^72^, ^74^, ^76^, ^146^
		^1^H MRS + ^31^P MRS	int	^32^, ^33^, ^55^, ^127^, ^138^, ^139^, ^140^
		^13^C MRS + ^31^P MRS	int	^66^, ^129^, ^130^
	Mouse	^1^H MRI + ^31^P MRS	int	^59^, ^136^, ^137^
	Rabbit	^1^H MRS + ^31^P MRS	sim	^27^
Brain	Human	^1^H MRS + ^31^P MRS	int/sim	^30^, ^35^, ^65^, ^71^
		^1^H MRI + ^23^Na MRI or ^2^H MRSI	int/sim	^28^, ^68^, ^70^, ^92^
	Cat	^1^H MRS + ^31^P MRS (+ ^23^Na MRS (+ ^19^F MRS))	int	^46^, ^47^, ^128^
		^19^F MRI + ^17^O MRI	int	^45^
	Rat	^1^H MRS or ^1^H MRI + ^31^P MRS or HP ^13^C MRS	int/sim	^63^, ^142^, ^148^, ^149^
Lung	Human	^1^H MRI + ^3^He (+^128^Xe MRI)	int	^36^, ^37^
	Rat	^1^H MRI + ^19^F MRI	int	^95^
Knee	Human	^1^H MRI + ^23^Na MRI	int/sim	^31^, ^48^, ^134^
	Rabbit	^1^H MRI + ^19^F MRI	sim	^132^
Liver	Rat	^1^H MRS + ^31^P MRS	sim	^44^
		^1^H MRI + HP ^13^C MRS	sim	^142^
Kidney	Mouse	^1^H MRI + HP ^13^C MRI	sim	^67^
Breast	Human	^1^H MRI + ^23^Na MRI	syn	^69^
Heart	Human	^31^P MRS + ^1^H pencil navigators	int	^90^, ^91^
Whole body	Mouse	^19^F MRI + ^1^H MRI motion correction	sim	^94^
	Rabbit	^19^F MRI + ^1^H MRI motion correction	sim	^93^

### 5.1 Skeletal muscle

The vast majority of interleaved multi‐nuclear papers so far published are reports of studies performed in skeletal muscle. The explanation is twofold: first, skeletal muscle is the organ that experiences by far the fastest and greatest physiological and metabolic adaptations upon activation, and only interleaved acquisitions are capable of monitoring multiple physiological variables quasi‐simultaneously, which is necessary to study their interactions with sufficient temporal resolution. Second, limb investigation is much less constraining in terms of spatial localization, which simplifies coil setup and sequence design.

The first interleaved ^1^H/^31^P MR study of human skeletal muscle investigated the effect of hypoxia during an incremental knee‐extension exercise, monitoring in parallel intramyocytic oxygen partial pressure (PO_2_) calculated from the deoxymyoglobin (dMb) desaturation level, the high‐energy phosphates and intracellular pH in the quadriceps.^127^ The main contribution of the study to exercise physiology however was through integration of dMb‐derived intramyocytic PO_2_ with invasive determination of blood flow, arterial and venous PO_2_ to determine for the first time the O_2_ diffusional conductance at intermediate muscle O_2_ consumption ( ${V˙}_{O_{2}}$).

**FIGURE 4:**
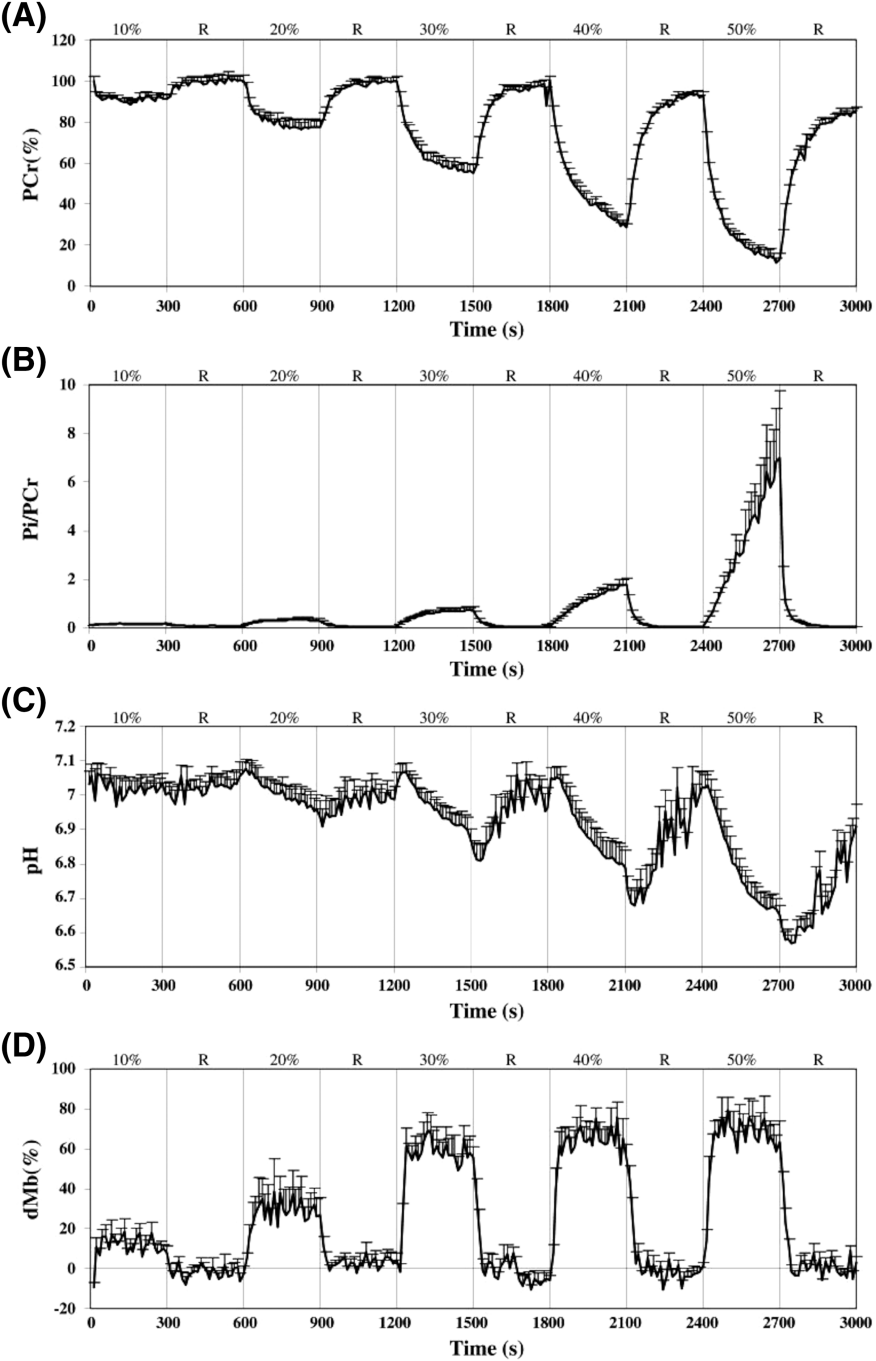
Mean + SE curve obtained from interleaved ^1^H/^31^P MRS acquisitions during the different force steps (10, 20, 30, 40 and 50% of quadriceps maximal isometric voluntary torque) and the subsequent recoveries (R) of the voluntary contraction exercise session for PCr (A), [P_i_]/[PCr] ratio (B), pH (C) and dMb (D). Figure reproduced with permission from Reference^140^

Also during an incremental knee‐extension protocol with a very similar ^1^H/^31^P setting (see Figure 4), electrically stimulated muscle contractions were compared with voluntary contractions.^140^ While it was confirmed that energy requirements were much higher for electrical stimulation contractions to generate the same work as voluntary contractions, it was also observed that for an identical inorganic phosphate (P_i_) to phosphocreatine (PCr) ratio, [P_i_]/[PCr], the dMb level was less elevated, showing that if anything the O_2_ supply‐to‐demand ratio was rather improved. This was compatible with earlier ^15^O_2_ and H_2_^15^O positron emission tomography studies, which had shown massive vasodilation and hyperperfusion induced by electrical stimulation in parallel with the O_2_ consumption increase associated with this less efficient mode of motor unit recruitment.^150^ Recent work done in the finger flexor muscles where near‐infrared spectroscopy measurements were added confirmed that dMb is a major contributor of the near‐infrared spectroscopy signal in muscle.^138^

Interleaved non‐localized ^13^C/^31^P MRS has also been performed to study the glycogen synthesis rate dependence of insulin resistance simultaneously with glucose‐6‐phosphate (G6P), an intermediate in glycogen synthesis, following a 20 min‐long exercise.^130^ During the insulin‐independent phase (first hour after exercise), no differences in G6P concentration and glycogen synthesis rate were found between insulin‐resistant offspring of parents with non‐insulin‐dependent diabetes mellitus with respect to age‐matched healthy subjects, whereas glycogen synthesis rate was lower in the patients during the insulin‐dependent phase (second to fifth hour of recovery). In contrast, no statistically significant difference of the mean G6P concentration was found, despite being systematically lower in the control group. This technique was also used in a separate study,^66^ performed in healthy subjects, showing an increase in G6P concentration and glycogen synthesis during the first 15 min after heavy exercise but a reduced glycogen resynthesis rate for several hours in muscle with high glycogen concentration, suggesting an inhibiting feedback mechanism of glycogen in its resynthesis. A major step forward occurred with the addition of a perfusion imaging module to the ^1^H/^31^P non‐localized MRS sequence.^32^ In the first studies, skeletal muscle perfusion was measured with an MR version of venous occlusion plethysmography, which was rapidly replaced by a more efficient pulsed ASL variant (SATIR).^151^ A spin echo blood oxygenation level dependent (BOLD) signal reflecting capillary oxygenation was also possible to obtain with SATIR. This sequence was used to investigate a number of conditions, as described in the following paragraphs.

In the field of exercise physiology, differences in skeletal muscle energy metabolism and perfusion control were documented between endurance and sprint athletes. Evidence was collected linking Mb concentration and energy metabolism efficacy.^76^ On the assumption that arterial O_2_ content and mitochondrial oxidative coupling are normal, it was shown that multi‐nuclear interleaving during the recovery phase of a plantar flexion bout could provide O_2_ supply, uptake and consumption rates in the calf from ASL perfusion values, ^1^H Mb resaturation and creatine rephosphorylation rates, respectively. By gathering these elements within the same physiological stress, the oxygen extraction rate could be calculated.^72^

In relation with aging, it was demonstrated that in healthy elderly subjects the perfusion response to aerobic exercise was somewhat reduced as compared with young adults, but no difference in maximum mitochondrial ATP production was observed.^141^ However, during the exercise bout itself, adenosine diphosphate control of oxidative phosphorylation appeared to be slightly but significantly impaired. In a subsequent study, acute administration of an antioxidant cocktail was shown to improve both perfusion and mitochondrial ATP production during exercise recovery in the elderly subjects only.^77^

The interleaved sequence was also able to reveal previously unidentified pathological mechanisms in Type 3 glycogen storage disease. In addition to a defective debranching enzyme activity, the patients have abnormal muscle perfusion response to moderate exercise. Combined analysis of dMb, BOLD, perfusion and PCr curves during exercise recovery concluded a role of perfusion in the lower ATP production, on top of the enzyme deficiency, that might contribute to the phenotype shift of the disease from childhood to adulthood.^74^ More anecdotally, the aetiology of abnormally low mitochondrial ATP production in mitochondrial diabetes and peripheral artery disease patients^72^ was characterized using this sequence. More recently, this interleaved sequence was implemented on a 3 T clinical scanner, without the need of any hardware modifications from the user side,^60^ other than employing a dual‐tuned ^1^H/^31^P RF coil. The repeatability of the multiparametric acquisition during an ischaemia–hyperaemia paradigm and a plantar flexion exercise bout was assessed, while taking into account visit‐ and subject‐specific nOe effects. In both paradigms, negative correlations were found between $T_{2}^{*}$ and pH during recovery and, at the end of exercise, the PCr depletion correlated with the percentage of Mb desaturation.

**FIGURE 5:**
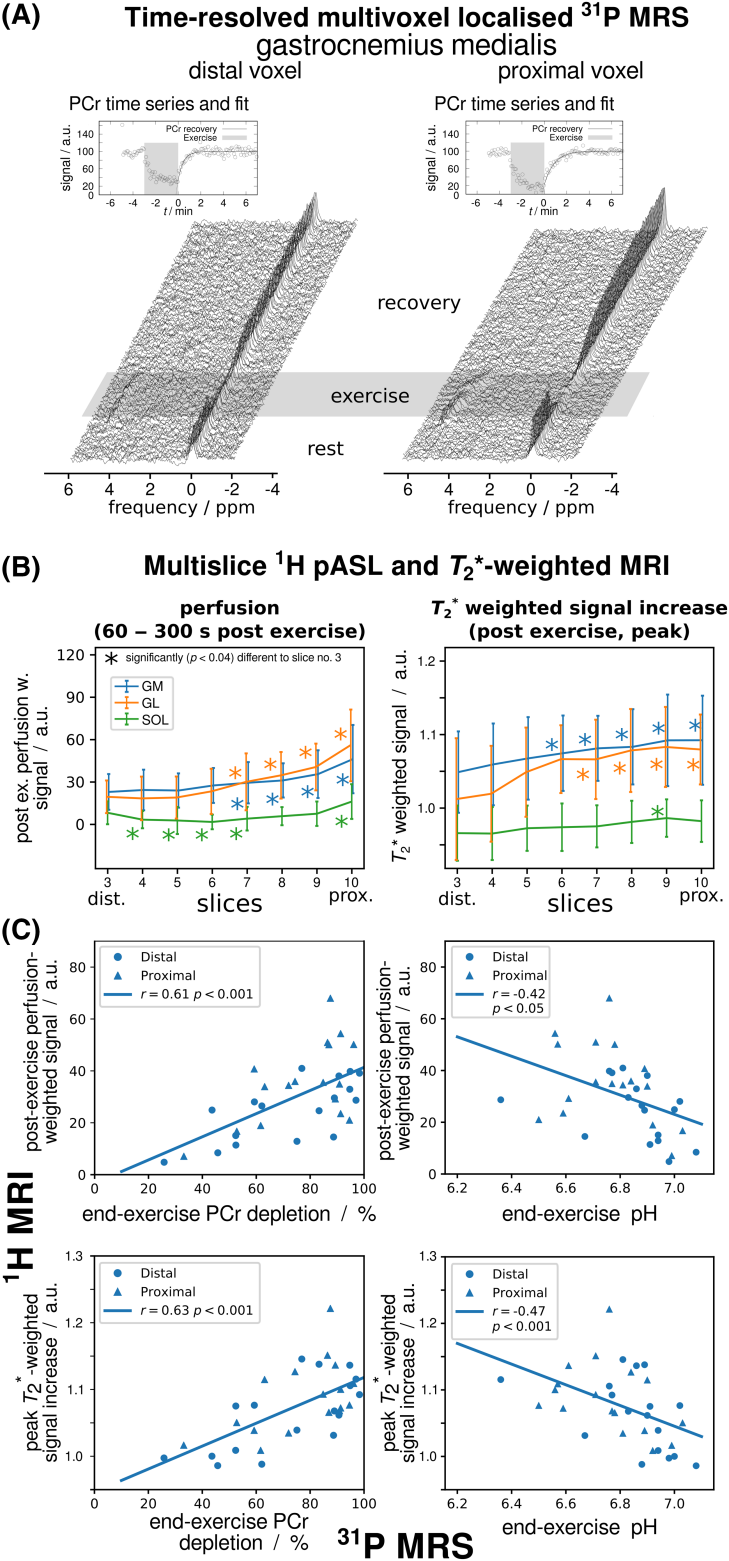
A, B, Time series of localized ^31^P MR spectra from two adjacent positions in gastrocnemius muscle (A) were acquired interleaved with multi‐slice pulsed ASL ^1^H MR images covering the same volume, providing tissue blood perfusion and $T_{2}^{*}$‐weighted images (B). Stronger PCr depletion and pH drop were found proximally rather than distally with ^31^P MRS, while at the same time stronger perfusion and $T_{2}^{*}$‐weighted signal increases were found with ^1^H MRI in the more metabolically active proximal regions of gastrocnemius muscle. C, Stronger end‐exercise depletion was associated with stronger acidification and upregulated perfusion. Figure adapted from Reference,^34^ which is licensed under CC‐BY‐4.0

A sequence performing ^1^H $T_{2}^{*}$ mapping and adiabatic pulse–acquire ^31^P MRS was used on peripheral artery disease patients, finding a negative correlation between PCr recovery rate and the BOLD amplitude during hyperaemia.^144^ The sensitivity available with a 7 T human scanner was invested into improving spatial information of interleaved measurements by implementing, for the first time, multi‐slice pulsed ASL in combination with multiple ^31^P semi‐LASER voxels^34^ placeable at arbitrary positions.^54^ Two ^31^P spectra were acquired from the gastrocnemius muscle every 6 s (Figure 5A), while perfusion and $T_{2}^{*}$ contrast were measured in 10 slices (Figure 5B). The study showed that metabolic activity, which was recently found to vary significantly along a single muscle,^152^ was tightly coupled to haemodynamic changes measured during the same exercise: the significantly higher end‐exercise PCr depletion, stronger pH drop and slightly elevated PCr recovery times were positively correlated with perfusion and $T_{2}^{*}$ changes measured in the gastrocnemius (Figure 5C).

Studying acid–base metabolism and glycolytic control requires concurrent quantification of ^31^P MRS‐visible high‐energy metabolites and lactate (Lac), which has ^1^H resonances that overlap with much stronger lipid resonances in muscle. A sequence interleaving non‐localized ^1^H and ^31^P spectroscopy with ^1^H double quantum filtered (DQF) MRS for Lac detection^32^ was implemented at 3 T. Repeatability and feasibility were demonstrated in the tibialis anterior muscle during ischaemic dorsi‐flexion exercise in healthy subjects.^32^ After ordering effects dominating the appearance of Lac resonances in anisotropic muscle tissue had been discovered,^153^, ^154^ ^1^H and ^31^P STEAM‐localized spectroscopy was interleaved with localized ^1^H DQF, taking muscle orientation into account.^55^ In this work, absolute quantification of Lac and phosphorylated metabolites was achieved in situ, during and following ischaemic plantar flexion exercise. Despite the complexities of Lac quantification in the presence of lipid signals^155^ and in anisotropic tissue,^153^, ^156^ the method showed excellent agreement of estimated [Lac] with values quantified ex vivo. Consistent results from interleaved direct pH and [Lac] measurements and from indirect analysis of proton handling confirmed assumptions on cytosolic buffer capacity in vivo.^55^ The concept of dynamic investigations using interleaved multi‐nuclear measurements was also adapted for small‐animal skeletal‐muscle applications at 4 T.^59^

Myostatin inhibition causes an increase in muscle mass, but compromised force production has been reported in isolated mstn^(‐/‐)^ muscle. Exerting the interleaved dynamic protocol on mstn^(‐/‐)^ mice revealed a reduced oxidative mitochondrial capacity, a reduced BOLD contrast (indicating a possible decrease in oxygen extraction) and a prolonged hyperaemia response with respect to wild‐type mice. Additionally, an increased proportion of Type IIb fibre and an unaltered capillary density were observed with histology, leading to the conclusion that the mstn^(‐/‐)^ model has a non‐pathologic shift towards glycolytic metabolism.^136^

The effect of electropermeabilization was evaluated on muscle function using an empty plasmid 15 d after electropermeabilization, considered as the end of the regenerative phase. Interleaved measurements showed altered perfusion and bioenergetics in electropermeabilized mice, whereas histological findings demonstrated a decreased number of Type IIb fibre but increased capillary density and number of Type I and IIa fibres. Although a decrease in 10% of cross‐sectional muscle area was found, the specific muscle force did not change.^137^

### 5.2 Lung

During the past decade, interleaving of ^1^H, ^3^He and ^129^Xe imaging has been developed for lung studies. The different diffusivity and solubility properties of ^3^He and ^129^Xe MR provide complementary information on ventilation, perfusion and lung microstructure, while ^1^H MRI provides anatomical and functional data.^22^, ^157^ Interleaving enhances the complementarity of these methods by acquiring the datasets within the same physiological state, reducing spatial mismatches caused by variations in lung inflation or diaphragm position, and shortens the required breath‐hold duration. Furthermore, acquiring ^1^H MRI anatomical data simultaneously with ^3^He or ^129^Xe images would allow their co‐registration with anatomical CT images, the clinical gold standard in diseases, such as emphysema and cystic fibrosis, and in lung radiotherapy.

**FIGURE 6:**
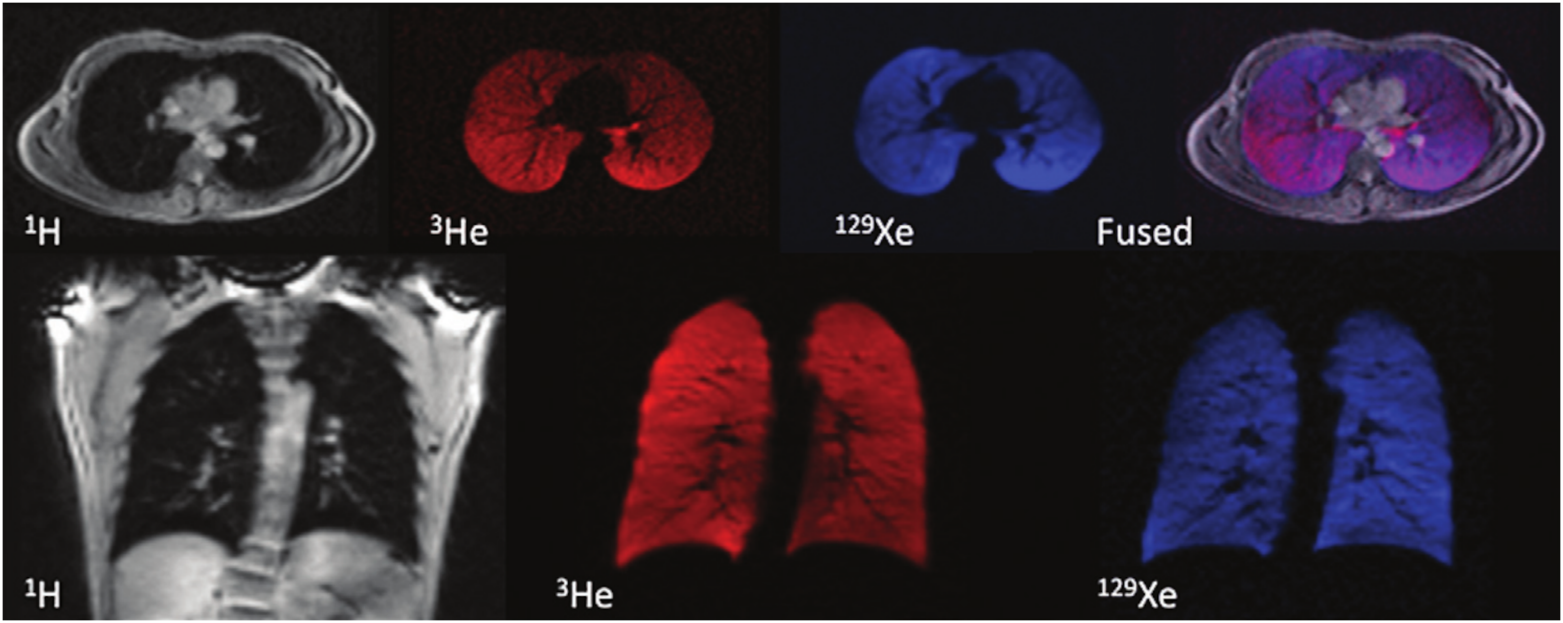
MR images of ^1^H (grey), ^3^He (red) and ^129^Xe (blue) acquired from a healthy volunteer in the same breath‐hold containing 600 mL of ^129^Xe and 300 mL of ^3^He. The anatomic ^1^H images show excellent spatial registration with the ^3^He and ^129^Xe ventilation images, as demonstrated by the overlaid fused image (purple). Figure reproduced with permission from Reference^37^

Wild et al^36^ performed interleaved ^1^H and HP ^3^He MRI in vivo at 3 T using the scanner's ^1^H quadrature body coil and a linear ^3^He Helmholtz coil, each coil actively detuning while the other one was active. GRE images of both nuclei were acquired during a 15 s breath‐hold, in healthy subjects and in a patient afflicted by lung cancer and chronic obstructive pulmonary disease. When the ^1^H and ^3^He images were acquired in separate breath‐holds, the ventilation volume overlap between repeated breath‐holds was 87.4% and 86.7% for a volunteer and the patient, respectively. In the patient, despite the effort to replicate the breath‐hold manoeuvre, misregistration was always visible. The authors noted that by interleaving the measurement of individual phase encoding lines of the ^1^H and ^3^He images (5 ms gap), motion misregistration errors were further reduced by limiting the effect of cardiac pulsatility. This work was later extended^37^ to include HP ^129^Xe imaging using a setup of electrically isolated RF coils comprising a flexible ^129^Xe quadrature vest transceiver inside an elliptical ^3^He birdcage coil nested inside the ^1^H body coil (Figure 6). The ^3^He and ^129^Xe coils' tunings were verified while nested and with the load of a volunteer. By taking advantage of the different diffusivities of ^129^Xe and ^3^He, dual‐gas imaging could be used to enhance detection of partial obstructions in the same inflation state while the ventilation volumes would be provided by the ^1^H anatomic images.^37^

A triple‐tuned RF coil with improved ^1^H reception was later created by the same group for 1.5 T use, although interleaved acquisitions were limited by the requirement of the new coil to manually activate the T/R switch of the nuclei.^158^

Studies employing ^1^H and ^19^F MRI simultaneously with retrospective motion correction were performed in rabbits,^93^ mice^94^ and rats.^95^ The 6% larger field of view of ^1^H images, originating from the gyromagnetic ratio differences, was compensated during image post‐processing.^94^ Lowering voxel resolution for increased SNR on ^19^F images was obtained by applying a spherical weighting to the image k‐space, reducing its radius.^94^, ^159^

### 5.3 Brain

Reductions of scan time and improvements of SNR per unit time have been achieved with interleaved acquisitions in human brain,^30^, ^71^ in particular when interleaving 2D ^1^H MRSI with 3D ^31^P MRSI. SNR per unit time increased by 12% and 80% for the ^31^P and ^1^H datasets, respectively, for the same duration (50 min) compared with non‐interleaved, serial acquisitions.^65^ ^1^H MR fingerprinting and ^23^Na MRI^70^ has also been carried out at 7 T (Figure 2). At 4 T, fluid‐attenuated inversion recovery (FLAIR) images (14 slices) were acquired over 7 min while interleaving with ^2^H MRSI ( 13×9×11 matrix, spherical encoding, 2 averages), 60 min after an oral intake of [6,6'‐^2^H_2_]‐glucose.^68^

Non‐localized interleaved ^1^H and ^31^P MRS has been performed in the hypoxic cat brain and the ischaemic mouse brain to monitor concentration changes of ATP, PCr, P_i_ and Lac as well as intracellular pH changes.^63^, ^128^, ^148^ In the mouse, PCr and ATP had completely depleted 10 min after the arterial occlusion. At the end of the 30 min ischaemia, Lac concentration had increased 10‐fold ( 15.8±2.5 μ mol/g) and pH had decreased from 7.14±0.01 to 6.32±0.10. About 1 h after reperfusion, metabolite concentrations had returned to baseline levels. A linear regression analysis showed a strong correlation (−0.97) between intracellular pH and [Lac].

Interleaved ^1^H PRESS (alternating between 30 ms and 136 ms $T_{E}$ for Lac detection) and ^31^P slab‐selective pulse–acquire spectroscopy measurements were made at 4 T in five awake humans during hypocapnia. During the 20 min‐long hyperventilation period, a (modest) maximum increase in pH (0.047) occurred at the 14th minute, maximum Lac accumulation was reached 1 min later and only minor PCr (−3.4*%*) and P_i_ (+6.4*%*) changes were observed.^35^ At the end of the 20 min recovery period, the partial pressure of carbon dioxide, pH and P_i_ had not recovered to pre‐hypocapnia values. The modest changes observed during hyperventilation, contrasting with studies carried out in anaesthetized animals, suggested an adaptive response of human brain to hyperventilation or a deregulation of cerebrovasculature under anaesthesia.

Taylor et al^149^ explored the ischaemic rat brain with an interleaved ^1^H PRESS/^31^P FID sequence. The interleaving measurements revealed that PCr responses to occlusion were quite similar between subjects, as previously thought, but the Lac responses showed higher inter‐individual variability. By comparing the Δ([Lac]/[PCr]) ratio, two rat subgroups could be differentiated. The authors hypothesized that the low Δ([Lac]/[PCr]) value was an indicator of a reduced metabolic reserve of glucose and glycogen. Again, a strong correlation was found between [Lac] and pH (−0.85) during the 12 min ischaemia.

A study carried out in cats with interleaved ^17^O and ^19^F MRSI aimed to evaluate the cerebral metabolic rate of oxygen consumption (CMRO_2_) in a 0.8 cm^3^ voxel in the parietal cortex. While breathing a gas mixture of ^17^O_2_ and CHF_3_, CBF was measured with the inert CHF_3_ tracer while CMRO_2_ was estimated from the H_2_^17^O concentration (above the natural abundance value) in the voxel and the measured CBF using a single‐compartment model. From the seven studied animals, a wide variation of CBF and CMRO_2_ was observed, but a good correlation between CBF and CMRO_2_ was also found.^45^

The study of metabolite kinetics using HP ^13^C in an organ is affected by non‐specific signal contributions arising from vascular and extracellular compartments. To circumvent this difficulty, an injection of HP ^13^C‐labeled pyruvate, administered intravenously, followed by a gadolinium‐based CA was performed on the rat to isolate the signal from the intracellular compartment in the brain and liver. The pyruvate and Lac dynamics were then evaluated using a two‐compartment model.^142^ Performing simultaneous ^1^H and ^13^C MRI allowed inclusion of the $T_{1}$ variability (from the CA concentration) as an additional model parameter.

In humans, retrospective motion correction of ^23^Na MRI was recently performed^92^ using ^1^H 3D navigator image volumes. Navigator data had a temporal resolution of 6 s and matched the spatial resolution of the ^23^Na data. Both ^23^Na data consistency between consecutive scans and image quality were improved.

### 5.4 Kidney

Simultaneous spectral–spatial Cartesian ^1^H and spiral HP ^13^C MRI acquisitions were used to track pyruvate and Lac dynamics in the kidney during free breathing. Every 5 s, two ^1^H images of water and fat, a 1D ^13^C spectrum used to measure the relative frequency of pyruvate and two ^13^C images of Lac and pyruvate were acquired. The ^1^H images were used to retrospectively compensate for motion during region‐of‐interest (ROI) positioning and to discard motion‐corrupted images.^67^

### 5.5 Heart

Cardiac studies in humans have employed interleaved acquisitions for ^1^H image navigation to compensate for respiratory motion in ^31^P spectra. The effectiveness has been demonstrated using pencil‐beam shaped ^1^H excitations at 1.5 T^90^ and has also been implemented with image‐based navigators at 7 T.^91^ In rabbits, motion correction was implemented by simultaneous ^1^H and ^19^F MRI for imaging of the heart.^93^

### 5.6 Knee

In a study in the knee,^31^ the acquisition time of four different 3D datasets including ^23^Na images with or without the contribution of long‐ $T_{2}$ components, a ^1^H $T_{2}^{*}$ map and a three‐point Dixon (GRE multi‐ $T_{E}$ acquisition) was halved, resulting in a total scan time of 23 min and 25 s when acquiring in interleaved mode. Another study^48^ also halved the acquisition time by simultaneously acquiring 3D UTE radial ^1^H and ^23^Na images.

### 5.7 Breast

In breast imaging, simultaneous ^1^H and ^23^Na MRI reduced the acquisition time by half at 3 T.^69^

## 6 PERSPECTIVES FOR CLINICAL APPLICATIONS

The added value of multi‐nuclear interleaving in combination with decreasing technical difficulties for its implementation, notably in the clinical setting, are incentives for the MR community to further invest in this methodology. The design and benefits of interleaved and simultaneous pulse sequences will nevertheless depend on the application, organ of interest and availability of a dual‐tuned coil that fulfils the required sensitivity and spatial coverage.

Based on studies using classical ‘sequential’ sequences, a few multi‐nuclear applications are briefly discussed below and are summarized in Table 3. Furthermore, in the specific case where the individual datasets acquired during multi‐nuclear interleaving present very different VOIs,^32^, ^55^, ^160^ volume‐specific $B_{0}$‐shimming configurations could greatly improve data quality.^161^, ^162^, ^163^

**TABLE 3:** Multi‐nuclear MR applications that may benefit from being implemented as simultaneous and interleaved protocols

Target	Application	Gain	Multi‐nuclear methods
Oncology	Improved tumour characterization and monitoring	Reduced acquisition time	^1^H + ^31^P MRS
Brain	Metabolic profiling and quantification	Reduced acquisition time	^1^H + ^31^P MRS/MRSI
	Richer examination in bipolar disorder (Li concentration, membrane turnover, pH and Mg^2+^)	Reduced acquisition time	^7^Li + ^31^P MRSI
	Motion correction for long 3D ^7^Li MRSI acquisitions in bipolar disorder	Improved data quality	^7^Li MRSI + ^1^H navigator
Muscle	Faster ^1^H and ^31^P examinations in neuromuscular diseases	Reduced acquisition time	^1^H MRI + ^31^P MRS/MRSI
	Discrimination of alkaline P_i_ resonances in dystrophic muscle	Reduced acquisition time	^1^H + ^31^P MRS
	Blood flow and energy metabolism evaluation in individual muscles	Multiparametric information	^1^H MR + localized ^31^P MRS
	Simultaneous measurement of IMCL, glycogen and G6P synthesis and storage following exercise	Multiparametric information	^13^C + ^31^P MRS, ^13^C + ^1^H MRS
Heart	Motion correction of localized ^31^P MRS	Improved spectral quality	Localized ^31^P MRS + ^1^H navigators
	Measurement of metabolic biomarkers (CK reaction, [PCr], [ATP])	Reduced acquisition time	^1^H MR + localized ^31^P MRS
Lung	Evaluate gas uptake and transfer times with anatomical or perfusion information	Multiparametric information	^129^Xe MR + ^1^H MRI
	Continuous ventilation imaging in normoxia conditions with anatomical or perfusion information	Multiparametric information	^19^F + ^1^H MRI
Liver	Combined fat fraction, IHCL and ^31^P MRS measurements in NAFLD and NASH	Reduced acquisition time	^1^H MR + ^31^P MRS
	Motion correction from breathing in ^31^P MRSI acquisitions	Improved data quality	^1^H navigator + ^31^P MRSI
Cartilage	Inclusion of ^23^Na imaging for improved detection of osteoarthritis and cartilage repair monitoring	Reduced acquisition time	^1^H + ^23^Na MRI
Bone	Complementary quantitative mineral bone content and bone matrix density values for improved diagnosis	Reduced acquisition time	^1^H UTE or ZTE + ^31^P ZTE

**Abbreviations:** IMCL, intramyocellular lipid; CK, creatine kinase; IHCL, intrahepatocellular lipid; NAFLD, non‐alcoholic fatty liver disease; NASH, non‐alcoholic steatohepatitis.

### 6.1 MRS in brain and oncology

MRS has demonstrated its usefulness in classifying mass lesions and tumours and in monitoring their therapeutic treatment.^12^ The complementary information brought by ^1^H MRS alone during an MRI examination increased the rate of correct diagnoses by 15.4%.^164^ ^31^P MRS has also been used, to a lesser extent, for tumour classification and monitoring.^12^, ^165^, ^166^, ^167^, ^168^ Interleaving ^31^P MRS with ^1^H MRS could prove a useful application in oncology and in numerous brain studies by extracting complementary metabolic information within clinically feasible time constraints.^29^, ^30^, ^71^

In bipolar patients under lithium administration, interleaving ^7^Li and ^31^P MRSI could simultaneously provide ^7^Li levels in tissue and information on free Mg^2+^, pH and cell membrane anomalies.^169^, ^170^) Moreover, long X‐nucleus acquisitions, such as ^7^Li 3D MRSI (46 min, Reference^170^), could benefit from ^1^H navigators for movement correction.^81^

### 6.2 Muscle

Evaluations in neuromuscular diseases typically include fat infiltration, muscle water $T_{2}$, lean mass and muscle cross‐sectional area measurements using ^1^H MRI. ^31^P MRS is also included in mitochondrial myopathies, congenital lipodystrophy, muscular dystrophies and fibromyalgia.^171^, ^172^ Cellular membrane damage and 'leakiness' in dystrophic muscle has also been evaluated by comparing ^1^H‐ and ^31^P‐based pH values.^173^ Interleaving ^1^H MRI (or MRS) and ^31^P MRS could provide in these diseases a reduction in acquisition time. Glycogen detection by ^13^C MRS could also be combined with ^31^P or ^1^H MRS after a physical effort to simultaneously evaluate glucose transportation and phosphorylation, glycogen synthesis and the changes of lipids and glucose storage and utilization with respect to exercise and diet.^66^, ^129^, ^174^

During a transient state, such as exercise, dynamic acquisitions interleaving fast multiparametric imaging schemes such as vPIVOT or SAGE^160^, ^175^ with localized ^31^P single‐voxel spectroscopy (SVS)^33^, ^34^, ^176^ could provide a more detailed evaluation of energy metabolism and oxygen consumption in individual muscles.^72^, ^177^ Translation of DQF Lac MRS to ultra‐high field^178^ has the potential to further increase sensitivity of interleaved ^1^H/^31^P measurements^55^ to study acid–base metabolism and glycolytic control. By reducing the temporal resolution, fast MRSI modalities could replace the localized SVS module.^57^, ^179^

### 6.3 Lung

Dynamic ^129^Xe MRS yields information on the surface‐to‐volume ratio and gas transfer times, while ^129^Xe MRI explores ventilation, regional gas uptake^180^ and alveolar‐capillary exchange.^181^ ^19^F MRI has also been used for ventilation imaging under normoxic conditions at high temporal resolutions.^182^, ^183^, ^184^ The value of such ^129^Xe or ^19^F MR datasets could be enriched by interleaving them with anatomical^185^ or perfusion^186^ information by ^1^H MRI during single breath‐hold or continuous ventilation.

### 6.4 Heart

Localized ^31^P MRS provides relevant biomarkers (CK reaction, [PCr], [ATP]) in cardiomyopathies, diabetes, heart failure, aortic stenosis after valve replacement and during exercise paradigms.^187^, ^188^ Interleaving ^31^P MRS with ^1^H MRI sequences is a viable clinical option as the individual datasets have similar acquisition lengths ( ∼ 10 min for gated ^31^P MRSI at 1.5 or 3 T^189^, ^190^). Other than examination length reduction, interleaving could enable navigators during ^31^P MRS, potentially increasing data quality and repeatability.

### 6.5 Liver

^31^P MRS(I) has been used to asses regenerative activity^191^, ^192^ and to evaluate graft function following transplantation.^193^

Localized ^31^P MRS could benefit from MR navigators by reducing the impact of breathing, whereas interleaving ^31^P MRSI with ^1^H MR, for monitoring intrahepatocellular lipids or fat‐fraction values, could reduce total scan time. These tools could constitute interesting clinical applications in prevalent diseases such as non‐alcoholic fatty liver disease and non‐alcoholic steatohepatitis.^194^

### 6.6 Cartilage

Changes in sodium concentration, an indirect measure of glycosamine sulfate proteoglycan (GAG) content, is evidence of early osteoarthritis and correlates with cartilage repair.^195^ In this context, ^23^Na imaging could be interleaved with ^1^H MRI for morphological^196^ or comparative information (UTE $T_{2}^{*}$, gagCEST^197^, ^198^) at a reduced total examination time and without requiring CAs.

### 6.7 Bone

^1^H UTE and zero echo time (ZTE) imaging provides information on the density and mechanical properties of bone matrix whereas high‐resolution MRI has been used for microarchitecture imaging in trabecular bone,^199^, ^200^ albeit at clinically long acquisition times. ^31^P MRI can provide mineral content information at the cost of long acquisition times ( ∼ 20 to 37 min^9^, ^10^, ^201^). The repetition times used in bone ^31^P ZTE MRI ( ∼ 150 ms) could be used for interleaved ^1^H ZTE and high‐resolution acquisitions for a reduced examination length. Combining bone matrix density and mineral content information could allow differentiation of osteoporosis from demineralizing disorders, a necessity for accurate diagnosis, intervention and monitoring of clinical responses to treatment.

## 7 CONCLUSION

Interleaved and simultaneous multi‐nuclear MR acquisition protocols have a wide range of applications, from reduction of total acquisition time to improved X‐nucleus data quality by adding ^1^H‐derived dynamic adjustments to multiparametric acquisitions within a single dynamic experiment, granting insights that are difficult or impossible to obtain by other means. While some early and experimental systems allowed for such measurements relatively straightforwardly, this became increasingly difficult on clinical MRI scanners in the past and required specific hardware modifications. Fortunately, the latest generation of MR systems of major vendors removed this hardware limitation, enabling interleaved or simultaneous multi‐nuclear acquisition provided that the system supports X‐nucleus measurements and a dedicated dual‐tuned coil is available. The dual‐tuned RF coil plays an important role, and multiple designs exist for optimal performance in a specific organ and application. The significant added value of interleaving for clinical applications and research, accompanied by the decreasing technical difficulties for its implementation, are major incentives to further invest in and standardize interleaved and simultaneous multi‐nuclear acquisitions. While most results have been obtained in muscle, promising non‐proton MR applications are abundant throughout different organs, particularly due to increasing sensitivity of MR systems, and new multi‐nuclear MR applications can be envisaged to increase the value of clinical MR.

## Data Availability

Data sharing is not applicable to this article as no new data were created or analyzed in this study.
